# New Advances in Tissue Metabolomics: A Review

**DOI:** 10.3390/metabo11100672

**Published:** 2021-09-30

**Authors:** Michelle Saoi, Philip Britz-McKibbin

**Affiliations:** 1 Department of Chemistry and Chemical Biology, McMaster University, Hamilton, ON L8S 4M1, Canada; saoim@mskcc.org; 2 Donald B. and Catherine C. Marron Cancer Metabolism Center, Memorial Sloan Kettering Cancer Center, New York, NY 10065, USA

**Keywords:** review, capillary electrophoresis, cancer, chromatography, clinical medicine, metabolomics, mass spectrometry, nuclear magnetic resonance, sample preparation, tissue biopsies

## Abstract

Metabolomics offers a hypothesis-generating approach for biomarker discovery in clinical medicine while also providing better understanding of the underlying mechanisms of chronic diseases. Clinical metabolomic studies largely rely on human biofluids (e.g., plasma, urine) as a more convenient specimen type for investigation. However, biofluids are non-organ specific reflecting complex biochemical processes throughout the body, which may complicate biochemical interpretations. For these reasons, tissue metabolomic studies enable deeper insights into aberrant metabolism occurring at the direct site of disease pathogenesis. This review highlights new advances in metabolomics for ex vivo analysis, as well as in situ imaging of tissue specimens, including diverse tissue types from animal models and human participants. Moreover, we discuss key pre-analytical and post-analytical challenges in tissue metabolomics for robust biomarker discovery with a focus on new methodological advances introduced over the past six years, including innovative clinical applications for improved screening, diagnostic testing, and therapeutic interventions for cancer.

## 1. Introduction: A Historical Perspective to Tissue Metabolomics

Renowned French pathologist and founder of modern histology, Marie François Xavier Bichat, first defined the term “tissue” as fundamental structural units of organs in the human body, comprised of interlaced vessels and fibers resembling a woven structure [1]. Bichat’s tissue doctrine of general anatomy stated that the analysis of tissue specimens was critical for understanding the origin of human diseases, as well as the development of effective therapeutic interventions [1,2]. Through his contributions, histology emerged as a systematic study of tissue specimens using microscopy. In 1847, Kün [3] first reported the use of a needle-biopsy technique for extracting tumor tissue for microscopy. Thereafter, Sir James Paget and Sir John Erichsen [4,5] subsequently introduced a needle-aspiration biopsy method for histological analysis of breast tumors. The needle biopsy technique was further developed as a diagnostic tool for assessing tumors later in the 20th century. For instance, the needle aspiration biopsy technique became a routine clinical practice at the Memorial Center in New York, where 2500 tumors were reported over a three year period in the mid-1920s [5,6]. While initially being rejected in the US, the needle-aspiration biopsy technique continued to be used across Europe. Ultimately, within the latter half of the 20th century, further developments in the technique led to its widespread clinical use in other countries worldwide.

At present, tissue biopsies are routinely used in histology and cytology for the diagnosis of malignant from benign tumors. Despite the prevalent use of histology in modern clinical medicine, the method is prone to observer bias that stem from visual (e.g., differences in illusion of size, brightness, color hues) and cognitive biases (e.g., confirmation and/or context bias) that may lead to subjective interpretation and grading when using semi-quantitative scoring systems [7]. For instance, Kleiner et al. [8] performed a study to examine inter- and intra-observer variability in histopathology scoring of liver specimens from patients with non-alcoholic fatty acid liver disease (NAFLD) as they are at high risk for developing cirrhosis and hepatocellular carcinoma (HCC) if left untreated. While all pathologists showed high agreement in steatosis and fibrosis scoring among adult cases, there was less agreement in pediatric cases, especially when evaluating the presence of microvesicular steatosis, pigmented macrophages and ballooning [8]. While histopathology and semi-quantitative grading may be useful for patient stratification in diseases such as NAFLD, there is growing use of non-invasive imaging techniques [9] and biochemical tests [10] that are less prone to bias to ensure more reliable clinical decision making.

Tissue biopsies have emerged as a clinical specimen of interest in “-omics” approaches, including genomics, epigenomics, transcriptomics, proteomics and recently metabolomics [11]. Metabolomics aims to characterize and quantify low molecular weight metabolites (<1.5 kDa) within complex biological samples when using high field nuclear magnetic resonance (NMR) and various separation techniques coupled to high resolution mass spectrometry (MS), which can provide new insights into disease mechanisms [12,13]. Nontargeted metabolite profiling can also reveal the mechanisms of action of therapeutic interventions and adherence monitoring, including exercise training and/or dietary changes [14,15]. Traditionally, most metabolomics studies utilize surrogate biofluids (e.g., urine, plasma) due to their less invasive sample collection procedures, which are especially useful for biomonitoring applications in large populations. However, biofluids are non-organ specific and are reflective of many biochemical processes occurring over various tissues within the body [16]. As a result, metabolic phenotyping of tissue specimens is ideal (if available) as they are the localized site of most disease processes relevant to organ dysfunction in specific disease processes, such as chronic kidney disease, coronary artery disease, and inflammatory bowel disease. Furthermore, they may provide more robust and sensitive biomarkers for disease screening, diagnosis or prognosis as compared to blood or urine specimens notably at early stages of development [17].

**Figure 1:**
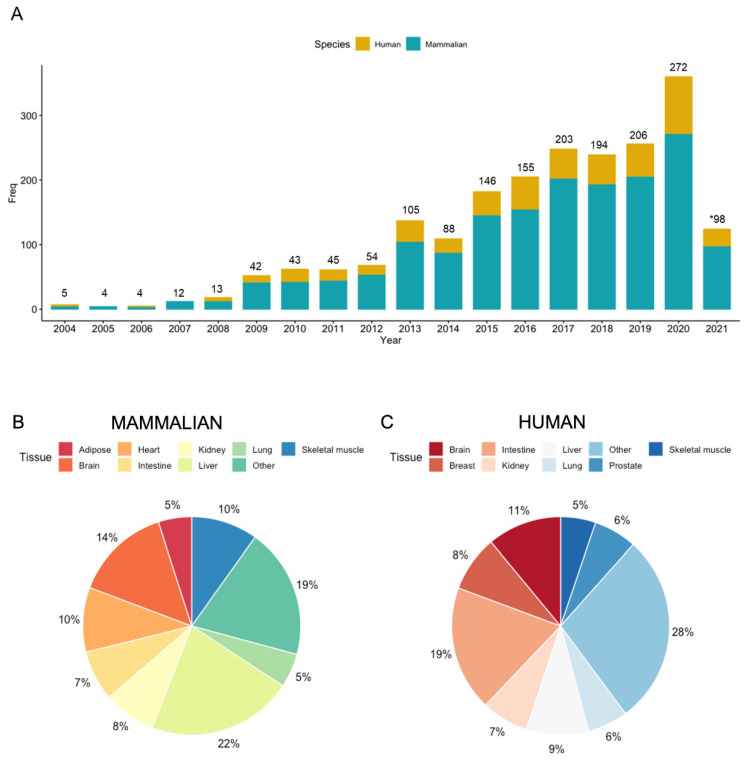
(**A**) An overview of published research articles in the field of tissue metabolomics from January 2002 until May 2021 (*) based on a PubMed search with the terms “tissue metabolomics.” Only original research articles were reported (excluding comprehensive reviews, book chapters, commentaries, conference abstracts) based on mammalian species (blue). Approximately 30% of these studies were based on human tissue specimens (yellow). Pie chart distributions of various (**B**) mammalian and (**C**) human tissue specimens analyzed from these published studies.

Within the last two decades, there has been a steady growth in tissue metabolomic applications, including new advances in sample preparation, as well as instrumental and bioinformatic methods to improve the identification of clinically relevant metabolites and lipids from minimal amounts of tissue [18]. For example, Watkins et al. [19] reported in 2002 one of the first comprehensive tissue metabolomic studies applied to murine heart and liver tissues to examine the effects of rosiglitazone on lipid metabolism in type 2 diabetes. In a recent PubMed search using the terms “tissue metabolomics,” over 1600 studies have been reported for mammalian tissue specimens from January 2002 to May 2021, with a majority of studies published from 2015 onwards as shown in Figure 1A. Moreover, a diverse range of tissue specimens have been analyzed in these published reports with liver (22%), brain (14%), heart (10%), skeletal muscle (10%) and kidney (8%) tissues comprising of the top five organs mainly analyzed from mammals in pre-clinical animal studies as highlighted in Figure 1B. Other mammalian tissues less frequently explored include adipose, pancreas and breast tissue. Approximately 30% of these studies involved tissue specimens collected from human participants—the most common being intestine (19%), brain (11%), liver (9%), breast (8%), and kidney (7%) tissue specimens as indicated in Figure 1C. A majority of these studies employed global metabolomic approaches for differentiating tumor-related metabolite signatures in cancerous relative to noncancerous tissue, including HCC, colorectal cancer (CRC), and breast cancer, whereas brain tissue metabolomic studies have focused on neurodegenerative diseases, such as Alzheimer’s disease. As a result, tissue metabolomics is a rapidly expanding field in clinical medicine relevant for the prevention and/or treatment of chronic human diseases. This is important given an alarming increase in cancer burden globally with fatalities mainly attributed to lung, followed by colorectal, liver, stomach, and female breast cancers [20].

## 2. An Overview of Tissue Metabolomic Workflows

**Figure 2:**
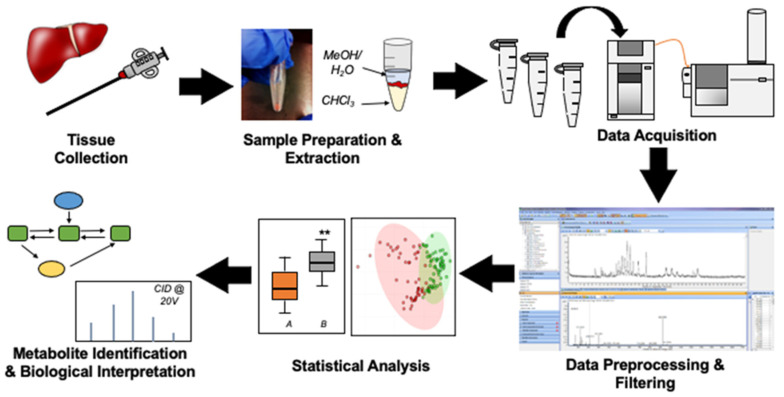
Overview of a classic data workflow for tissue metabolomic studies. Tissue collection via biopsy or autopsy by a trained physician represents the first key step in the workflow. Thereafter, tissue specimens undergo several steps of sample preparation (e.g., lyophilization, homogenization etc.) prior to sample extraction. Following rigorous sample preparation methods, metabolite profiling is performed using a suitable analytical platform of choice such as NMR, direct infusion and/or MS-based instrumental platforms with high resolution separations. Data preprocessing and filtering are then applied to the raw metabolome dataset to reduce dataset redundancy, spurious signals and false discoveries prior to statistical analysis, metabolite identification, and biological interpretation. Direct and spatially-resolved metabolomic analysis from tissue specimens is also feasible using new advances in MS imaging techniques.

To date, two complementary strategies have been adopted for tissue metabolomics studies, namely targeted (i.e., hypothesis-driven) analysis of known metabolites and/or non-targeted (i.e., hypothesis-generating) analysis of metabolites when using one or more instrumental platforms [21]. Targeted metabolomics traditionally focuses on measuring a defined subset of defined metabolites within one or more metabolic pathways of interest in order to answer a defined biochemical question [12]. However, quantitative metabolomic analyses of increasingly large metabolite panels are now feasible when using high throughput NMR [22], and notably direct infusion-MS methods using suitable matching stable-isotope internal standards [23] that also enables rapid spatial imaging of lipid profiles directly from tissue specimens [24]. In general, data preprocessing, statistical analyses, and biochemical interpretation is more routine in targeted metabolomics than non-targeted metabolite profiling data workflows [25] given that a large fraction of the metabolome constitutes unknown compounds [26]. However, a major drawback of targeted approaches is their limited metabolite coverage that may be unsuitable for basic and applied discovery-based research [27]. In contrast, non-targeted metabolomics is a global approach that aims to measure a far wider range of metabolites within a tissue specimen, including the identification of unknown metabolites of biological or clinical significance when using MS/MS [28]. In contrast to targeted approaches, non-targeted metabolomic studies aim to filter, authenticate, and annotate metabolites from potentially thousands of molecular features detected when using full-scan data acquisition with high resolution MS, whose chemical structures are largely unknown a priori. High dimensional data sets are generated in tissue metabolomics, and thus require multivariate statistical methods for improved data visualization, pattern recognition/filtering, group classification, and metabolite ranking [29]. As chemical standards and stable-isotope internal standards are often not available, only semi-quantitative or relative quantification is feasible in discovery-based tissue metabolomics [30]. There are numerous steps involved in non-targeted tissue metabolomic workflows including pre-analytical, analytical and post-analytical processes (Figure 2) in order to obtain high data quality while reducing bias and false discoveries. These steps will be outlined in detail in the proceeding sections.

## 3. Tissue Collection and Sample Preparation

**Figure 3:**
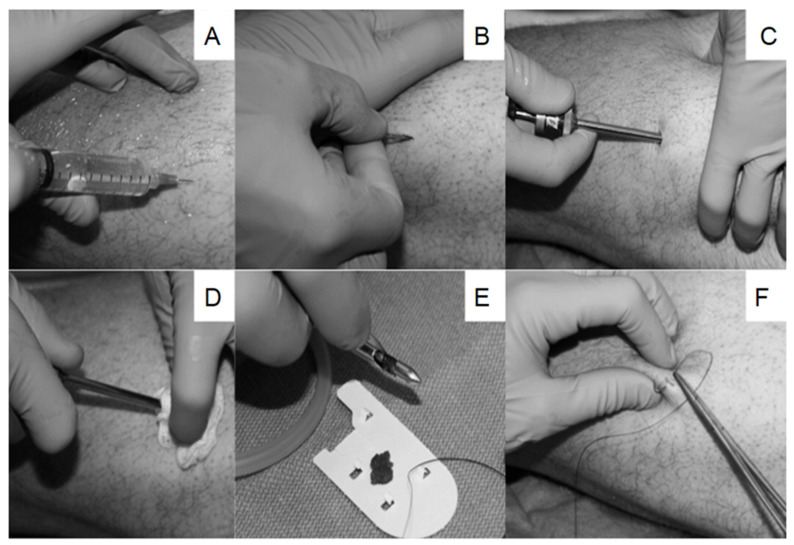
Schematic of a suction-modified Bergström muscle biopsy technique. (**A**) Lidocaine is first applied to the skin and subcutaneous tissue. (**B**) An incision is made though the subcutaneous tissue into the fascia to help guide the biopsy needle (**C**) into the muscle, where suction is applied to obtain the tissue specimen. (**D**) Removal of the needle is facilitated using counterpressure and twisting motion. (**E**) The specimen is examined for adequacy and dissected into smaller sections prior to flash freezing in liquid nitrogen. (**F**) The incision is closed using a 3.0 silk suture. Reproduced from Tarnopolsky et al., 2011 with permission.

Tissue collection and sample preparation remains the major bottlenecks and sources of bias in tissue metabolomic workflows. Tissue collection is usually performed under anesthesia through a biopsy, surgical procedure, or a post-mortem autopsy by a trained physician. Depending on the tissue of interest, various biopsy procedures may be used for tissue collection and storage. However, the most common procedures include percutaneous biopsy, core needle biopsy (CNB) and fine needle-aspiration biopsy (FNAB). The percutaneous biopsy is a classic outpatient procedure used for obtaining liver tissue for assessing disease severity, diagnosis and/or treatment responses. The technique involves inserting a biopsy needle through the abdomen to obtain a liver biopsy. Complications such as localized discomfort and/or pain as well as mild hypotension can arise. However, only 1–3% require hospitalization for these complications [31]. CNB is a biopsy technique routinely used in clinical and/or research settings that employs a large core needle to obtain intact tissues for examination. The method requires local anesthesia and is prone to minor complications, including pain, discomfort, bruising and infection [32,33]. Figure 3 illustrates an example of a CNB technique (i.e., Bergström muscle biopsy) introduced by Tarnopolsky et al. [34] for obtaining skeletal muscle tissue. After local anaesthesia is applied, the biopsy needle is inserted through the subcutaneous tissue, fascia, and into the muscle, where suction is applied to obtain the tissue [34]. Similar CNB techniques have been utilized for other tissue types, including liver [35], breast [36] and lymphatic tissue [37]. FNAB is a minimally invasive, low cost and simple biopsy technique that requires no anesthesia to obtain small amounts of tissue using a small, fine needle [38]. FNAB has been used in breast [39], thyroid [40] and pancreatic [41] tissue for disease diagnosis and/or monitoring tumor progression. Nonetheless, tissue biopsies still remain an invasive procedure that requires informed patient consent, which limits its applicability to recruit large numbers of healthy volunteers as controls.

There are numerous other precautions one should take for collecting tissues for non-targeted metabolomics studies [30]. For instance, after tissue collection via biopsy or surgical procedures, tissues should be removed of fat and connective tissue as well as potential contamination from blood, in order to obtain an accurate, metabolite profile representative of the tissue. Moreover, it is imperative to immediately freeze tissues upon collection (i.e., flash-freezing in liquid nitrogen) and store at low temperatures (−80 °C) in order to halt metabolism and ensure metabolites within tissues remain stable with long-term storage [17,30]. This is crucial for labile metabolites such as major phosphagens (e.g., ATP) and reduced thiols (e.g., GSH) that are prone to hydrolysis and oxidation artifacts, respectively. These processes are exacerbated following repeat freeze–thaw analysis of frozen tissue specimens or their extracts [42].

After collection, tissues can either be lyophilized (i.e., freeze-dried) or remain wet prior to various sample workup protocols that are critical for subsequent metabolomic analysis, such as tissue disruption, liquid extraction, sample deproteinization and/or chemical derivatization. Certain tissues may require more extensive grinding with a nitrogen cooled mortar and pestle if needed, however, it is labor intensive and low throughput. As a result, tissue homogenization usually consists of physically disrupting the tissue using a homogenizer [43,44,45,46]. While homogenization is evidently less labor intensive, it is impractical for smaller tissue quantities [47]. Alternatively, lyophilization is recommended as an effective strategy to eliminate excess water in heterogenous tissue specimens [17]. In this case, lyophilization of wet tissue specimens offers a useful sample preparation step to reduce biological variance in metabolomic studies, as well as facilitate sample handling (e.g., weighing as a fine powder) and enhance subsequent solvent extraction efficiency. Reported metabolite concentrations are thus normalized to total dried tissue weight [42], which is allows for better comparative analysis of other heterogenous clinical specimens, such as loose stool collected from pediatric inflammatory bowel disease patients [48]. Therefore, several factors such as minimum tissue amount, total number of samples, and available infrastructure/budget must be considered when choosing an appropriate method for homogenization of tissue specimens prior to metabolite extraction.

To date, numerous metabolite extraction methods have been reported for various tissue specimens from animals and humans; this stems from the diverse polarity of metabolites and lipids that span a wide dynamic range having different solubilities and chemical stabilities [49]. As a result, there is no single extraction procedure that allows for truly unbiased tissue analyses while also being compatible with NMR and MS-based metabolomics [50]. In general, the optimal extraction method should be non-selective, reproducible, simple, and produce high metabolite yields with good long-term stability that can also be automated for large-scale studies [17]. Lin et al. [47] reported a simple, reproducible extraction method for muscle and liver tissues for NMR metabolomics. Various extraction solvents were compared in this study including perchloric acid, as well as organic solvents (i.e., methanol, ethanol, acetonitrile) mixed with water and/or chloroform. Overall, the methanol/chloroform/water solvent system based on the classic biphasic Folch and/or Bligh-Dyer extraction procedure was ideal due to its efficiency in obtaining both hydrophilic metabolites and non-polar lipids with high efficiency and reproducibility [47]. To date, many studies have made progress in overcoming the low throughput in tissue metabolite extraction methods that can be implemented in multi-platform approaches [16,46,49]. Furthermore, recent efforts have been made to establish standard extraction procedures for various tissue types such as liver, kidney, and skeletal muscle in murine tissues [16]. However, there is urgent need for better harmonization of various tissue metabolomic/lipidomic protocols similar to recent initiatives introduced for serum/plasma [50] and cell cultures [51].

## 4. Instrumental Methods for Tissue Metabolomics

Comprehensive analysis of complex and heterogeneous tissue specimens with high data fidelity remains a persistent challenge in metabolomics [52,53]. In most cases, complementary extraction conditions and/or analytical platforms are required to achieve broad metabolome coverage for a diverse range of compounds ranging from abundant electrolytes and osmolytes to lower abundance metabolites and lipids. Recent advances in both nuclear magnetic resonance (NMR) and mass spectrometry (MS)-based instrumental platforms enable the identification and quantification of metabolites especially when coupled to high efficiency separation techniques, such as gas chromatography (GC), liquid chromatography (LC), capillary electrophoresis (CE) and ion mobility (IM) [54]. Nevertheless, the development of a compatible multi-platform pipeline for reliable analyses of specific tissue specimens is important for optimal metabolome coverage [11].

Solution NMR spectroscopy offers a fast and reproducible platform for metabolite profiling and metabolic flux analysis [55] while allowing for quantitative and qualitative determination of metabolites with excellent long-term stability [56]. Moreover, minimal sample preparation is typically required allowing for the analysis of metabolites non-destructively in complex biological samples within minutes (i.e., ~10 min/sample) depending on spectral acquisition settings and magnet field strength. However, one-dimensional proton (^1^H)-NMR methods for tissue extracts generally require larger sample volumes (i.e., >100 μL) while also being prone to spectral overlap and lower concentration sensitivity (e.g., detection limits >5 µM) as compared to MS-based techniques [57]. In most cases, effective removal of lipids and protein from biofluids or tissue extracts is needed to improve the quality of NMR spectra [58] allowing for reliable automated spectral processing [59]. The advent of High Resolution Magic Angle Spectroscopy (HRMAS)-NMR, first introduced by Cheng et al. [60] in 1996 enables intact tissue analysis (i.e., 10–20 mg) without homogenization, extraction or complicated sample processing [61,62]. More importantly, since the sample remains unaltered during analysis, other diagnostic tests such as histopathology can be performed on the same tissue specimen. Since 2004, HRMAS-NMR has been successfully applied for metabolomics analyses of various tissue types including brain, breast, lung, and pancreas [63]. In particular, HRMAS-NMR has been used to examine regional differences in metabolite profiles between cancerous and adjacent, non-cancerous tissue that could better inform treatment decisions [64,65]. However, the use of spectral binning of integrated peaks from the analysis of NMR-observable tissue metabolites typically includes contributions from many known or undefined metabolites, thus lacking specificity for biochemical interpretation [66].

MS-based techniques have been more widely applied in tissue metabolomics studies due to their higher sensitivity and wider dynamic range as compared to NMR. Moreover, contemporary MS instrumentation have greatly improved analytical performance (e.g., mass resolution, mass accuracy, scanning speeds) to reduce isobaric interferences to measure distinct metabolite or lipid species with lower detection limits. However, non-targeted MS-based approaches are still constrained by inconsistent reporting standards and several technical challenges, including quantitative reliability and structural elucidation of novel compounds [67]. Direct infusion (DI)-MS of crude tissue extracts offers a “separation-free” platform making it an attractive technique for high throughput screening, such as shotgun lipidomics [68]. In this case, lipid extracts comprised of non-aqueous solvent(s) prevents the introduction of highly saline tissue samples. Greater sample throughput (>1500 samples/day) for discovery-based metabolomics can be achieved by flow injection analysis (FIA) coupled to time-of-flight mass analyzers with excellent intrascan dynamic range [27]. However, DI or FIA-MS methods are prone to ion suppression due to matrix effects as well as lower specificity due to lack of resolution of isobaric and isomeric ions that can contribute to false discoveries. Additionally, unknown identification is challenging due to convoluted MS/MS spectra from co-eluting ions when compared to reference spectral databases without orthogonal retention, migration or ion drift time information [52]. However, selective chemical derivatization strategies of lipid classes or sub-classes can be applied in shogun lipidomic workflows to reduce interferences when analyzing complex sample extracts [69]. Nevertheless, large-scale studies using DI-MS require implementation of preventative maintenance protocols and robust data pre-processing approaches to reduce long-term signal drift and bias, such as intra- and inter-batch correction algorithms as demonstrated with cardiac tissue extracts [70].

**Figure 4:**
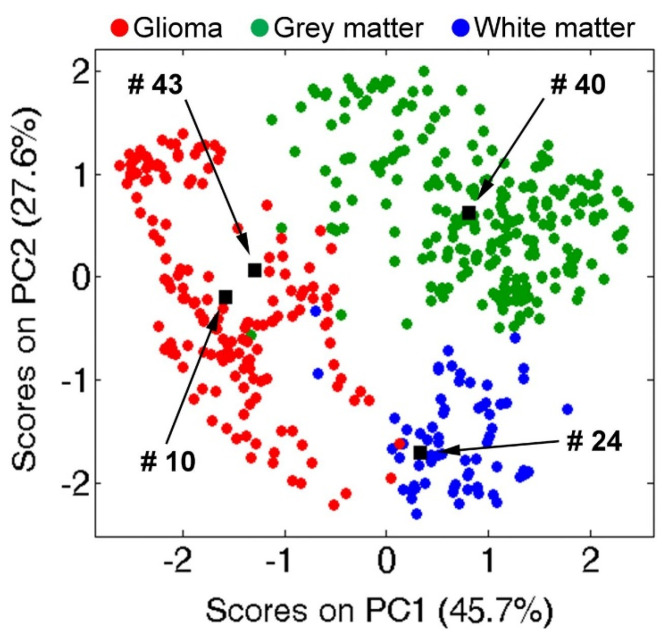
Chemical predictions of disease state for smears 10 (glioma, 99% TCP), 24 (white matter, 37% TCP), 40 (gray matter, 15% TCP), and 43 (glioma, 69% TCP). Projections of the tissue smears (black objects) are imposed on the principal component analysis (PCA) score space created from a reference DESI-MS spectral library; green, gray matter; blue, white matter; red, glioma. Adapted from Pirro et al., 2017 with permission.

Alternatively, ambient ionization-MS is a rapid technique for the direct analysis of tissue specimens with minimal sample preparation enabling ex vivo metabolic profiling without separation. The use of ambient MS-based techniques have increased for tissue metabolomics studies in recent years [71]. Desorption electrospray ionization (DESI)-MS, first introduced by Cooks and colleagues [72], enables the direct analysis of metabolites from intact tissue specimens by applying a fine spray of charged droplets to extract metabolites from the sample surface. Importantly, DESI-MS also allows for mass spectral imaging of tissue that retains the underlying spatial distribution of metabolite concentrations in situ that are otherwise lost with conventional metabolomic methods relying on tissue homogenization and extraction processes. Eberlin et al. [73] first developed a DESI-MS technique combined with machine learning methods for brain tumor classification based on lipidomic profiling in representative surgical specimens. Interestingly, the regional distribution of lipids shown in the DESI-MS images were highly correlated with the distribution of meningioma cells observed via histopathology of the same brain tissue specimen. Similarly, DESI-MS was also recently applied for rapid intraoperative assessment of tumor margins (<3 min) during glioma resection based on analysis of *N*-acetylaspartate, 2-hydroxyglutarate, and various membrane-based lipid profiles identified from brain tissue smears [74] as shown in Figure 4. This data provides prompt yet accurate information to better guide surgeons on safe tumor resection procedures on patients that is needed to reduce malignancy progression.

Probe electrospray ionization (PESI)-MS has also emerged as an ambient MS technique for real-time analysis of tissue specimens. The mechanism of ionization is achieved via insertion of an acupuncture needle into the sample of interest, where the water content enables ionization to occur upon application of a high voltage [75]. The advent of laser ablation electrospray ionization (LAESI)-MS by Nemes and Vertes [76] allows for both in situ and in vivo tissue analysis with no sample preparation over a wide mass range of ions. Using the native water content of the sample, a mid-infrared laser is focused on the tissue specimen, facilitating ablation followed by ionization [71]. LAESI-MS has been applied to various specimen types, most notably in brain tissue and single cells. Rapid Evaporative Ionization Mass Spectrometry (REIMS) or “iKnife” was recently introduced as a practical ambient ionization technique that also allows for in situ tissue sampling and real-time characterization of lipid profiles from human tissue to better guide surgical operations and procedures. In this case, a high frequency current is applied to the surgical blades to facilitate tissue plume formation to produce charged species that are subsequently removed by suction from the surgical site to a MS for data analysis [71]. Intriguing results reported by Balog et al. [77] revealed that the intraoperative REIMS technique coincided with postoperative histopathology results in 96.2% of reported cases, demonstrating the potential clinical utility of iKnife technology during surgical procedures. While the use of ambient MS-techniques is steadily increasing due to its ability to dynamically analyze spatial distributions of metabolites directly from tissue samples during operations, these techniques are still prone to matrix interferences and ion suppression effects. Moreover, a well-defined patient cohort is needed to validate differentiating lipid profiles associated with disease severity and heterogeneity in specific tissues during model training.

**Figure 5:**
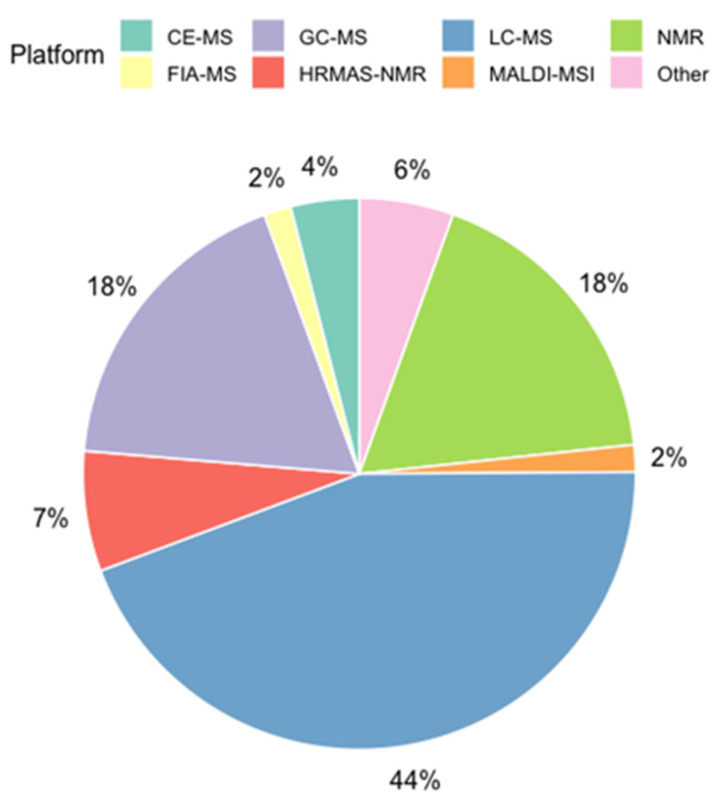
Representative pie chart summarizing the most common instrumental platforms used in tissue metabolomic studies published from January 2002 until May 2021.

To date, a majority of tissue metabolomics studies still rely on hyphenated analytical platforms that couple one or more separation methods to electrospray ionization-MS in order improve selectivity despite longer total analysis times and more complicated data pre-processing [56]. To date, tissue metabolomic studies have applied various instrumental configurations and data workflows based on targeted and/or non-targeted approaches as summarized in Figure 5. Overall, LC-MS is the most commonly used platform to date (~44%) due to the fact that complementary retention mechanisms can be used to resolve chemically diverse classes of metabolites and lipids from tissue extracts when using reversed-phase (RP) and hydrophilic interaction (HILIC) [78]. As a result, the broad selectivity of LC-MS enhances metabolome coverage with high sensitivity, small sample requirements, and compatibility with various mass analyzers [54]. GC-MS is the second most widely used analytical platform (~18%) in tissue metabolomics studies despite the need for more complex pre-column chemical derivatization and sample workup procedures, with most studies (~14%) using both LC-MS and GC-MS to increase overall metabolome coverage when analyzing tissue extracts [30,79,80,81]. Both solution NMR and HRMAS-NMR were used in about 25% of published tissue metabolomic studies as they are non-destructive methods that also allow for direct measurement of metabolites within intact tissue [57]. Interestingly, while CE-MS is ideal for the analysis of mass-restricted tissue specimens (<5 mg dried tissue mass), it remains an underutilized technique in tissue metabolomics (~4%) as compared to more established hyphenated-MS based platforms [42,82,83,84,85]. Furthermore, DI-MS and FIA-MS are also underrepresented instrumental platforms in tissue metabolomics studies (~1–2%) due to their low specificity and issues with ion suppression matrix effects and isomeric/isobaric interferences. Other emerging techniques recently used in tissue metabolomics include ultra-fast ion mobility-MS to improve the resolution and detection of low abundance lipids [86], as well as ambient MS techniques for spatially resolved tissue imaging applications as described above, including DESI-MS [87,88] and matrix-assisted desorption ionization (MALDI)-MS [89]. A list of representative metabolomic studies recently published are summarized in Table 1 that use different analytical methods applied to various tissue specimens.

## 5. Pre-Analytical Considerations to Reduce False Discoveries in Tissue Metabolomics

In order to achieve reproducible research findings with high data quality in non-targeted metabolomic studies, the utmost care must be taken in the pre-analytical phase to minimize false discoveries. Inadequate study power [90,91] is a major limitation in tissue metabolomics studies due to the modest number of study samples typically available, the large biological variance underlying disease heterogeneity, as well as the technical variability related to specimen collection, storage and workup. For these reasons, well designed studies should be performed, where comparison groups are closely matched in terms of anthropometric and clinical characteristics (i.e., sex, age, BMI, co-morbidities), especially when measuring small to modest effect sizes [92]. A unique advantage of tissue metabolomics is that each specimen can serve as its own control since specific malignant tissue regions can be analyzed relative to non-cancerous segments. This can be performed by resection of tissue segments prior to homogenization and extraction or directly via spatially resolved imaging MS techniques [93]. Furthermore, it is crucial to implement stringent quality control (QC) and quality assurance (QA) practices in the experimental workflow to minimize bias [94]. However, due to the diverse range of instrumental platforms, specimen types, sample workup procedures, and data workflows adopted in tissue metabolomics, a single unified QC/QA procedure may not fit all laboratories. Nonetheless specific guidelines are being increasingly adopted to promote good analytical practice and transparency in reporting, such as the Metabolomics Quality Assurance and Quality Control Consortium [95,96]. For instance, QA practices to prevent bias include implementation of preventative maintenance, regular instrument calibration, staff training in all laboratory operations, data storage and archival processes, as well as standardized operating protocols as related to tissue sample collection, extraction and storage. Similarly, QC procedures encompass analysis of suitable blanks, use of multiple internal/recovery standards, standard reference samples, and calibrants for reporting of data quality. However, the lack of certified standards for specific tissue specimens do not allow for effective harmonization studies as required to compare the performance of various analytical platforms between laboratories [97]. Alternatively, an internal reference material derived from pooled tissue samples in a study may serve as a surrogate QC sample for assessment of technical precision and long-term signal drift in longitudinal studies involving multi-user instrumentation.

## 6. Data Preprocessing and Statistical Analysis

Data preprocessing is a critical step for converting raw data prior to multivariate statistical analysis and metabolite identification [98]. This encompasses several steps including data filtering, peak picking and spectral deconvolution, time alignment, normalization and scaling [99,100]. In MS-based workflows, noise filtering is a crucial process that aims to authenticate molecular features from background ions (e.g., buffers, solvents), dataset redundancy (e.g., in-source fragments, isotopic ions, adducts) and spurious or irreproducible signals that constitute the majority of ions detected when using electron impact (GC-MS) or electrospray (LC/CE-MS) ionization with full-scan data acquisition [101]. This process is critical to eliminate redundant information in the data matrix while avoid data overfitting and false discoveries. Thereafter, an authentic molecular feature reflecting a unique metabolite can be annotated based on at least two orthogonal parameters, such as accurate mass and retention time (*m/z*:RT). Peak alignment is also a key data pre-processing step in tissue metabolomics to correct for long-term instrumental drift and potential isobaric interferences during data acquisition when using separation-based MS platforms. Additionally, authenticated molecular features in discovery-based metabolomic studies often need to satisfy specific criteria for acceptable technical precision based on repeat analysis of pooled QC specimens (CV < 35%), as well as their detection frequency to reduce missing values (>75%) notably when conducting longitudinal or large-scale studies [102]. However, specific thresholds used for data filtering in nontargeted metabolomics are dependent on the specific instrumental platform, metabolite abundance within tissue sample, as well as the availability of matching stable-isotope internal standards.

Thereafter, normalization and scaling methods are frequently used to reduce data variance while minimizing bias notably when comparing metabolite or lipid profiles over a wide dynamic range across different samples over time [96]. A common strategy in MS-based workflows is to normalize feature responses to internal standards, based on the assumption that systematic error exclusively contributes to the variance observed in the internal standards. For tissue metabolomics studies, feature responses are also often normalized to the (wet or dry) weight of tissue specimen analyzed, to account for variations in tissue specimens analyzed [42,85]. Mathematical transformations (i.e., log transformation) can also be employed to correct for heteroscedasticity and skewed distribution in the datasets [96]. Additionally, scaling methods (i.e., autoscaling, Pareto scaling, range scaling) are frequently used to correct for variances in feature abundances, where highly abundant features are scaled down to reduce their influence on statistical outcomes compared with features of lower abundance [103]. Furthermore, different strategies for missing value input are needed for non-detected metabolites measured in sub-sets of samples based on their impact of overall data variance and data quality, such as a k-means nearest neighbour algorithm [104]. To date, there are a plethora of open-source and commercial software packages and resources available for processing of metabolomic data sets [105], including XCMS, mzMine and MetaboAnalyst.

Prior to statistical analysis, the use of normality testing is warranted as most metabolomics data may be skewed and not normally distributed. Graphical methods (i.e., histograms) as well as univariate normality tests such as the Kolmogorov-Smirnov (K-S) or Shapiro-Wilk tests may be used in this case. However, if the data remains skewed, even after applying transformations, non-parametric tests must be used (i.e., Mann-Whitney U test) [90]. Multivariate statistical analysis methods are employed as a first step to evaluate the relationship (i.e., correlations, covariances) amongst all metabolites simultaneously in a dataset. Unsupervised methods such as principal component analysis (PCA) and hierarchical clustering analysis (HCA) are common exploratory methods used to assess the overall data structure while observing trends and groupings due to inherent variation without information on the data structure. In contrast, supervised methods such as partial least squares-discriminant analysis (PLS-DA) have a priori information regarding the data structure and thus, are often used to generate predictive models for classification. It is imperative to validate these predictive models using cross-validation and independent test set validation to decrease the risk of data overfitting and false discoveries [90,91]. Other methods such as bootstrapping, permutation testing and rotation tests can also be used for method validation in tissue metabolomic studies. For large, complex metabolomics datasets, univariate significance testing such as a paired or unpaired t-test are performed for tens to hundreds of metabolites simultaneously. In such cases, multiple hypothesis testing correction, such as the Bonferroni correction or the Benjamini-Hochberg false discovery adjustment is recommended to control false positives (type I errors) and false negatives (type II errors). Additionally, univariate statistical tests for robust biomarker candidates should also be adjusted for potential confounding following adjustments of covariates in a study (e.g., sex, age, BMI etc.) [106].

## 7. Unknown Compound Identification via High Resolution MS/MS

Unlike NMR, most detectable molecular features in MS based metabolomics remain largely unknown [26]. Indeed, a significant fraction of the human metabolome is derived from exogenous compounds derived from dietary intake, environmental exposures, and the microbiome [107]. As a result, no publicly available databases provide complete reference MS/MS spectra for all metabolites or lipids present in various tissues and species so reliance on *de novo* structural elucidation for chemical identification is often needed prior to biological interpretation. Contemporary software from MS vendors enable automated generation of most likely molecular formulae based on high resolution MS, including the accurate mass of a molecular ion, its isotopic pattern and charge state [108]. However, this is still insufficient to deduce a definitive chemical structure, yet can filter out potential isobaric candidates especially when combined with independent solute retention time, mobility or drift time information. As a result, collisional-induced dissociation experiments are often used for acquisition of MS/MS spectra from precursor ions. Recommended guidelines in the confidence of metabolite reporting is dependent on the specific application and compound class [109], with more stringent requirements for unambiguous lipid structural identification based on their unique stereochemistry [110]. Even if reference MS/MS spectra are available for a putative candidate ion in various curated spectral databases such as HMDB [111], METLIN [112], or LIPID MAPS [113], direct comparisons may be challenging due to co-eluting interferences without optimal spectral deconvolution approaches [114]. Alternatively, a global pathway meta-analysis approach directly from unidentified metabolite features may offer useful biochemical insights into mechanisms, such as in breast cancer [115].

Additional methods to support metabolite identification include functional group/chemical reactivity, enzymatic transformations, correlation analysis to other known metabolites, as well as integration with genetic data [116]. Furthermore, *in silico* approaches for predicting MS/MS spectra [117] in conjunction with target-specific databases relevant to species/sample type represent promising developments [118]. Some progress has also been made in establishing tissue specific databases, such as the Mouse Multiple Tissue Metabolome Database (MMMDB) introduced by Soga et al. [119]. The MMMDB is a curated public database that provides quantitative metabolite information from multiple murine tissues and plasma using non-targeted metabolomics approaches. The database contains annotated mass spectra and electropherograms that are readily accessible for on-line data comparison with other studies. Similarly, Fouroutan et al. [120] performed a comprehensive characterization of seven different bovine tissues (e.g., liver, muscle) and five different biofluids (e.g., serum, ruminal fluid) using NMR, LC-MS/MS and ICP-MS with references ranges reported for 2100 metabolites, lipids, electrolytes, and trace metals. These findings led to the creation of the Bovine Metabolome Database (BMDB), a public database and open access resource summarizing experimental, computational and literature research relevant to beef and dairy researchers, food/nutritional scientists, and consumers [120].

**Figure 6:**
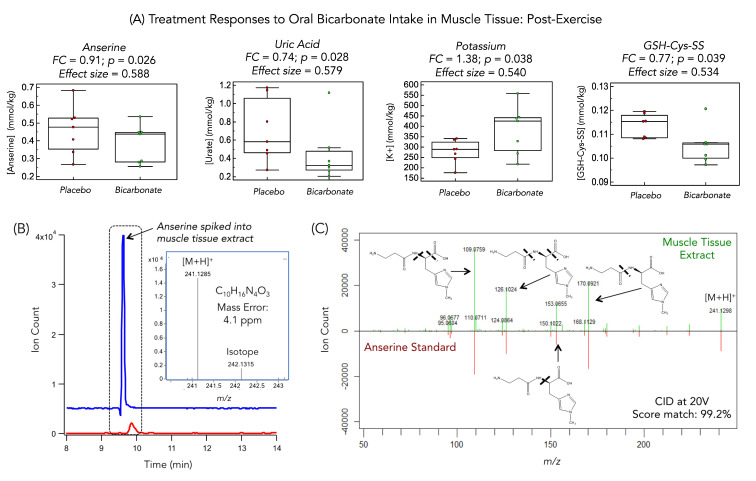
(**A**) Box-whisker plots of four top-ranked intracellular skeletal muscle metabolites/electrolytes (*p* < 0.05) associated with improved muscle function due to oral bicarbonate pretreatment following strenuous interval exercise in this placebo-controlled cross-over intervention study including an initially unknown metabolite at *m/z* 241.1295. (**B**) Extracted ion electropherogram with full-scan mass spectrum of the unknown ion, subsequently identified unambiguously as anserine by comigration after spiking pooled muscle tissue with an authentic standard. (**C**) Mirror plot comparing MS/MS spectra acquired for the unknown ion at 20 V as compared to anserine standard demonstrates a high matching score based on four characteristic product ions that is consistent with the β-alanyl-1-methylhistidine dipeptide. Adapted from Saoi et al., 2019 with permission.

Figure 6 illustrates a MS/MS spectra acquired for an unknown intramuscular cation (*m/z* 241.1295, [M + H]^+^) that was elevated following high dose oral bicarbonate pretreatment prior to strenuous exercise in a randomized placebo-controlled cross-over intervention trial [42]. The unknown cation was subsequently identified as anserine, a unique β-alanyl-1-methylhistidine dipeptide. This identification was achieved based on its co-migration after spiking a pooled muscle tissue extract together with excellent MS/MS spectral overlap as shown in the mirror plot with four diagnostic products ions that match well with a reference compound acquired under standardized conditions (20 V). Access to a purified standard is critical for confirmatory identification while also enabling its accurate quantification when reporting its intramuscular concentrations normalized to dried mass. Alternatively, a comparison of an unknown ion with a putative known analog based on differences in their characteristic MS/MS spectra and retention/migration time changes provides additional evidence for metabolite identification, such as 3-hydroxyhexanoylcarnitine and hexanoylcarnitine from murine placental tissue extracts [85]. Nevertheless, metabolite identification remains a significant bottleneck in global tissue metabolomic studies, notably when commercial standards and reference MS/MS spectra are lacking.

## 8. Applications of Tissue Metabolomics in Clinical Research: Recent Advances

Since tissues are the origins of aberrant metabolism from aging and/or environmental stressors (e.g., viral infections, carcinogen exposure etc.), they are ideal specimens for exploring the mechanisms underlying disease pathogenesis, such as advanced stages of liver fibrosis from chronic hepatitis C infection [121]. One of the most illustrative tissue metabolomics studies was the discovery of sarcosine as a putative biomarker of prostate cancer in 2009 [122]. Elevations in sarcosine were observed in metastatic and clinically localized prostate cancer tissues compared to benign tissues (*n* = 42), and these changes were also associated with cancer progression in patients with matching serum (*n* = 110) and urine (*n* = 110) samples. These results indicated that sarcosine could serve as a potential biomarker for early detection of prostate cancer given limitations of screening using digital rectal exams and prostate specific antigen tests. However, there have been subsequent studies reporting poor associations between sarcosine levels and prostate cancer progression in both urine [123] and serum [124], suggesting its limited utility for prostate cancer screening. Additionally, de Vogel et al. [125] reported that high serum sarcosine levels were modestly associated with reduced prostate cancer risk, contrary to the original findings by Sreekumar et al. [122]. These discordant findings highlight the need for independent replication while avoiding potential study design flaws, such as a temporal lag between serum collection and tissue biopsy sampling. Indeed, better standardization of biomarker validation studies, specimen collection procedures, and robust analytical protocols are required to render metabolomic discoveries more translatable in a clinical setting [126]. We will discuss recent tissue metabolomic studies with a focus on unique tissue specimens analyzed by innovative methods, as well as their application to improve clinical diagnostics for tissue-specific cancers lacking effective biomarkers for their early detection, such as urachal adenocarcinomas [127].

Saglik et al. [128] recently reported the first characterization of pterygium tissue, a fibrovascular mass that typically forms in the eye, often causing vision loss. Elevated levels of arginine, methionine, glycine and tyrosine were measured in pterygium tissue as compared to normal conjunctiva tissues. While this was the first study to perform metabolite profiling on this tissue type, a major limitation of the study was the modest metabolome coverage when using FIA-MS that focused on amino acids. In contrast, Leuthold et al. [129] developed and validated a comprehensive metabolomics and lipidomics workflow using LC-MS to characterize human kidney tissue derived from patients undergoing routine radical nephrectomy. Over 4177 metabolic and lipidomic features were detected using LC-MS in orthogonal reversed-phase and HILIC separations under both positive and negative ion modes. Furthermore, over 260 polar/ionic metabolites were annotated, including organic acids, amino acids, purines, nucleosides, monosaccharides, sugar alcohols and acylcarnitines. Major lipids from kidney tissue extracts were also analyzed in this study, including phosphatidylcholines, phosphatidylserines, and phosphatidylglycerols, as well as ceramides, glycosphingolipids, diacylglycerols and triacylglycerols. Furthermore, a cross-platform comparison was performed when analyzing tumor and non-malignant kidney tissue from clear cell renal cell carcinoma patients, which demonstrated good mutual agreement of their non-targeted LC-MS assay relative to a commercial Biocrates Absolute IDQ p180 targeted assay kit [129]. Independent replication of discriminating disease biomarkers by two or more analytical platforms is an effective way to reduce false discoveries in metabolomics [121] prior to their subsequent validation in larger/multi-center cohorts. A recent study by Sato et al. [130] also demonstrated the clinical utility of global metabolomic analyses for classifying disease progression and malignant status among clear cell renal carcinoma patients, which were correlated with clinicopathological factors, such as tumor volume, pathological T stage, presence of metastasis, and Furman nuclear grade. Anchoring specific metabolite signatures with previously validated blood-based biomarkers, well-established diagnostic algorithms (e.g., histopathology, imaging), and/or clinically meaningful outcomes provides greater credibility in the potential utility of tissue-based metabolomic discoveries.

To date, there have been several metabolomic studies on skin/epidermis tissue specimens, such as *ex vivo* analysis of the volatile metabolome from skin biopsies using head-space solid-phase microextraction with GC-MS to differentiate melanoma from benign naevi cases [131]. However, the characterization of dynamic changes in skin tissue following an acute injury has been unexplored. For the first time, Ashrafi et al. [132] reported the wound metabolome following skin tissue biopsies performed on four spots on the inner arm of volunteers in conjunction with skin microbiome analyses. Non-invasive headspace sampling was also performed using polydimethylsiloxane patches positioned at the wound site at three time points following a 28 day period of healing. As expected, fewer features (129 versus 346) were detected in headspace wound sampling as compared to direct tissue biopsy analyses when using GC-MS. Temporal changes in metabolite levels were identified after a false discovery adjustment, where specific volatile organics from headspace sampling were associated with skin healing processes (e.g., blood flow), such as isobutyl-2,2,4-trimethyl-3-hydroxypentanoate. However, no changes in bacterial genera abundances were evident since they remain largely stable on the skin when normal healing processes occur after an acute injury [132]. Nevertheless, further validation of this study is warranted in a larger cohort with standardized sampling collection procedures to enable reliable quantification of metabolites from skin tissues/wounds, whereas MS/MS is needed for the identification of a large fraction of unknown volatile organic compounds.

**Figure 7:**
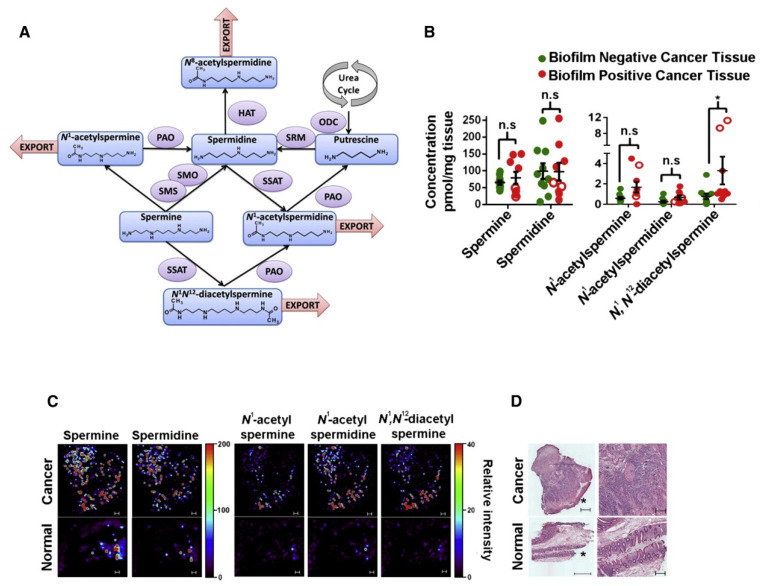
(**A**) Biofilm effects on polyamine metabolism in colon tissues, where PAO, polyamine oxidase; SSAT, spermidine/spermine N1-acetyltransferase; ODC, ornithine decarboxylase; HAT, histone acetyltransferase; SRM, spermidine synthase; SMO, spermine oxidase; SMS, spermine synthase. (**B**) Targeted metabolomics, concentrations of polyamine metabolites in cancers with (*n* = 9) or without (*n* = 10) biofilms (two-tailed Mann–Whitney test). Empty symbols indicate left-sided biofilm-positive samples. (**C**) Nanostructure imaging mass spectrometry on biofilm-positive normal and cancer tissue. Scale = 100 μm. (**D**) Hematoxylin and eosin staining, * mucosal edge. Scale = 500 μm left, 200 μm right column. Adapted from Johnson et al., 2015 with permission.

There have been numerous studies published on the characterization of human colon tissues given their key role in nutrient absorption and immunological adaptations especially in the context of colorectal cancer screening [133]. For example, previous studies have shown that right colon cancer patients have poor prognosis outcomes due to larger tumor sizes and higher tumor grade compared to those with left side colon cancer, which is exacerbated by obesity as a co-morbidity. Baxter et al. [134] used metabolomics to characterize differences between ascending and descending colon tissue from 24 adults scheduled for routine colonoscopy. Using an expanded yet targeted metabolomic analysis by LC-MS/MS, over 500 metabolites were detected and identified in both ascending and descending colon tissue biopsy extracts, including endogenous metabolites/lipids, as well as various dietary and microbiota-derived compounds. Overall, obese/overweight individuals had notable differences in their metabolome when comparing right-ascending and left-descending colon regions relative to normal weight controls, namely metabolites associated with gut inflammation, nutrient uptake, and products of microbiota metabolism, such as an enrichment in colonic trimethylamine-*N*-oxide [134]. The integration of metabolomic together with microbiome analyses of colonic mucosa may further improve understanding of microbiome dysbiosis that may also contribute to functional changes at specific cancer sites within the colon [135]. For example, Johnson et al. [136] used metabolomics to assess the impact biofilms have on colon tissue in the context of colon carcinogenesis. Using nontargeted LC-MS based metabolomics, over 300 molecular features were differentiated between cancerous and non-cancerous tissue from patients undergoing routine surgery from two sites at John Hopkins University (USA) and Karolinksa University Hospital (Sweden). Notable upregulations in polyamines including *N,N*-diacetylspermine, *N*-acetylspermidine, and *N*-acetylspermine were consistently observed in the two patient populations. Furthermore, Nanostructure Imaging Mass Spectrometry (NIMS) was used as a direct imaging method to confirm higher spatial distributions of polyamine concentrations in malignant tissue as compared to healthy controls, notably at the mucosal edge where biofilms typically form as depicted in Figure 7. Importantly, independent replication of discovery-based metabolomic analyses of colonic tissue extracts together with in situ imaging-MS and biochemical confirmation of increased spermidine/spermine *N1*-acetyltransferase expression provided supporting evidence that polyamine metabolites may be cooperatively produced by biofilm bacterial communities. These results revealed that targeting both polyamine production and biofilm interactions may pave the way towards a more successful therapeutic strategy in colon cancer [136].

There is growing interest in placental tissue metabolome analyses to explore the underlying mechanisms of early life exposures, and the developmental origins of disease in offspring since it functions as a critical interfacial organ during pregnancy. For instance, Walejko et al. [137] used NMR metabolomics to characterize both maternal and fetal placental tissue samples collected at two time intervals from non-labored women undergoing cesarian section following delivery. Overall, 40 metabolites were quantified consistently in these two distinct tissue types with a greater abundance of choline, as well as specific amino acids (e.g., Thr, Ser) and organic acids (e.g., citrate, succinate) in maternal as compared fetal tissue samples. Results from this study emphasize the maternal and fetal sides of the placenta have distinct metabolic phenotypes that likely reflects nutrient transport from the maternal to the fetal compartment via a concentration gradient during normal development [137]. Fattuoini et al. [138] also reported distinct differences in the placental metabolome from obese relative to normal weight pregnant women when using GC-MS, including metabolites associated with antioxidant defenses, lipid biosynthesis, and energy production. Furthermore, Saoi et al. [85] revealed sex-dependent differences in the placental metabolome, with intracellular metabolites associated with fatty acid oxidation and purine degradation were elevated in females as compared to male murine placentae. Obesity before pregnancy is associated with impaired metabolic status of the mother that impacts disease risk of the offspring [139], highlighting the important roles of habitual diet, maternal health, and early life chemical exposures that may have fetal sex-dependent susceptibilities.

## 9. Current Challenges in Tissue Metabolomics: Future Directions

Despite the mechanistic insights derived from tissue-based metabolomics studies in human participants and animal models, persistent technical challenges remain. These include the small amounts of tissue samples available for comprehensive cross-platform analyses, modest study power and inadequate control/patient specimens for rigorous biomarker validation, as well as the lack in harmonization of tissue-specific extraction procedures. Additionally, there remains few dedicated tissue biobanks for metabolomic/lipidomic studies, as well as public accessible MS/MS spectral libraries to support metabolite identification in specific tissues/species that include their reference concentrations. Due to the invasive nature of tissue biopsies in humans, limited sample quantities are typically obtained (~100 mg wet weight) yet often need to be utilized for other “-omics” platforms (e.g., transcriptomics, proteomics) or independent clinical tests (e.g., histopathology, cytology). Therefore, non-targeted tissue metabolomics studies face the challenge of achieving broad metabolite coverage while working with mass-limited tissue specimens. Although the majority of tissue metabolomic studies have been focused on differentiation of cancer from other types of malignancies or benign conditions, adipose tissue is not widely characterized despite its relevance in the pathogenesis of obesity-related chronic disorders, including diabetes and cardiovascular disease [140].

Recent studies have developed standardized workflows to overcome this challenge by maximizing the amount of metabolite information derived from the same tissue. For instance, Vorkas et al. [141] developed a pipeline for untargeted analysis using a simple extraction protocol to extract hydrophilic metabolites and non-polar lipids for cardiovascular disease research using the same arterial tissue with two consecutive extractions to improve metabolome coverage while using two orthogonal LC-MS methods (RP/HILIC) in positive and negative ion modes. This approach is applicable to other tissue metabolomic studies to achieve high data quality and broad metabolome coverage as reflected by 226 structural assigned metabolites and lipids identified from arterial tissue extracts [142]. Deeper metabolome coverage may be realized via chemical isotope labeling strategies for specific metabolite classes (i.e., sub-metabolomes) to enhance their retention properties and ionization responses when using LC-MS [142]. Huan et al. [143] reported one of the first studies to perform comprehensive metabolite profiling from intact tissue using a molecular preservation by extraction and fixation in conjunction with chemical isotope labeling and LC-MS, which enabled quantification of more than 4000 isotopic pairs of metabolites (i.e., ^13^C^/12^C-dansylated amine/phenol species) from stored prostate tissues. Importantly, this method enables the extraction of metabolites in methanol without alterations to tissue morphology, which allows histopathology or other clinical tests to be performed on the same tissue specimen. Alternatively, new advances in multiplexed CE-MS allows for higher throughput tissue metabolomic analyses, including novel data workflows for unambiguous biomarker classification via temporal signal pattern recognition [102]; this approach was used to identify a novel anserine analog reported for the first time from residual amounts of lyophilized human skeletal muscle tissue, where certain metabolites and electrolytes had strikingly higher relative abundances as compared their extracellular concentrations measured in fasting serum samples (e.g., phosphocreatine, reduced glutathione, potassium etc.) [42].

A major constraint of most tissue metabolomics studies published to date is the lack of spatial information, which is critical for accurately defining regions requiring surgical removal of malignant tissue. As a result, in situ localization of metabolites and their abundances in specific organelles or histologically defined parts of tissues is not feasible when using classical tissue extraction procedures or ex vivo tissue-based NMR approaches. Tissue metabolomic studies via imaging MS is thus ideally suited for analysis of the distribution of metabolites, lipids and/or drugs especially when combined with new advances in machine learning, deep learning, and artificial intelligence [144]. In fact, MS imaging heralds a revolutionary approach for digital pathology based on data-rich molecular information that may enable accurate discrimination of tumour from non-tumor regions of tissue after adequate model training [127]. However, further advances in tissue preparation, method reproducibility, faster acquisition times, and broader metabolome coverage is still needed. For instance, Li et al. [145] introduced air flow-assisted DESI-MS as a sensitive and more rapid approach for nontargeted and spatially resolved tissue metabolomic studies. This method allowed for a single imaging analysis of a lung cancer tissue cryosection (1 × 1 cm^2^) within 40 min as required for clinical lung cancer diagnosis and image-guided surgery. Lastly, the integration of microbiome, proteomic and/or genomic data sets is also critical in tissue metabolomic studies to better validate the putative clinical utility of prognostic or diagnostic biomarkers, as well as exploring their likely causative role in disease pathophysiology as demonstrated by the accumulation of hydroxybutyric acid metabolites in ovarian cancer [146]. This strategy can also lead to the identification of new potential therapeutic targets for treating cancers with poor survivorship, such as esophageal squamous cell carcinoma [147]. Nevertheless, future discovery-based tissue metabolomic studies are recommended to incorporate rigorous study designs that are replicated independently in representative populations using complementary analytical methods to demonstrate their reproducibility, diagnostic accuracy, and overall clinical utility based their estimated health impact [148].

## Figures and Tables

**Table 1:** Recently published metabolomic studies demonstrating advanced technical advancements in spatial imaging and/or mass limited analysis of various tissue specimens.

Analytical Platform	Tissue Type	Sample Size	Unique Features	Key Findings	Reference
LC-MS	Kidney	*n* = 5	Global analysis of metabolites and lipids by RP/HILIC	>1000 features reliably measured in kidney tissue with differentiation of malignant from non-cancerous tissue	Leuthold et al., 2017
LC-MS	Colon	*n* = 24	Analysis of ascending versus descending colon tissue	Colon lipids and metabolites elevated in obese/overweight as compared to normal weight with distinct regional differences in colon profiles	Baxter et al., 2020
LC-MS	Esophagus	*n =* 211	Validation of biomarkers of esophageal squamous cell carcinoma	Diagnostic/predictive metabolites with good accuracy that also provide insights into esophageal squamous cell carcinoma tissue calcification	Chen et al., 2021
GC-MS	Skin wound	*n* = 11	Novel tissue specimen and sampling method	Dynamic microbiome and metabolome analysis of >346 features during normal wound healing using patch sampling	Ashrafi et al., 2020
GCxGC-MS	Ovaries	*n* = 224	Predictive biomarkers of ovarian tumor burden and patient survival	Accumulation of hydroxybutyric acids with strong predictive ability of patient survival prior to surgery as confirmed by gene expression data	Hilvo et al., 2016
NMR	Placenta	*n* = 13	Novel tissue specimen from non-labored pregnancies	Differentiation of maternal and fetal placental tissue reflecting flux from mother to fetus following delivery	Walejko et al., 2018
NMR	Adipose	*n* = 3640	Visceral adipose tissue extract analysis in two large cohorts	Validation of a metabolite/lipid signature of visceral adiposity that persisted after adjustment for BMI	Neeland et al., 2019
HRMAS-NMR	Prostate	*n* = 365	Direct analysis of tumor grade and stage of prostate cancer	Differential analysis revealed metabolites were upregulated in tumor tissues with elevated *myo*-inositol	Vandergrift et al., 2018
DESI-MS	Brain smears	*n* = 73	Spatial imaging of tumor margins for resection	High tumor cell percentage at surgical margins with 93% sensitivity and 83% specificity for safe tumour resection	Pirro et al., 2017
DI-MS	Cardiac	*n* = 20	Best practice data workflows and rigorous quality assurance	8 batches of cardiac tissue extracts acquired over 7 days with inter-batch adjustment with QC spectra	Kirwan et al., 2014
MSI-CE-MS	Skeletal muscle	*n* = 14	Repeat muscle tissue biopsies in cross-over study using a multiplexed CE-MS platform	Modest treatment effect from bicarbonate intake prior to exercise with intramuscular changes in potassium, uric acid, oxidized mixed glutathione and anserine	Saoi et al., 2019
